# Growth rates of larval and juvenile bigeye scad Selar crumenophthalmus in captivity

**DOI:** 10.1186/2193-1801-2-634

**Published:** 2013-11-25

**Authors:** Aaron Welch, Ronald Hoenig, John Stieglitz, Zach Daugherty, Bruno Sardenberg, Sasa Miralao, Dan Farkas, Dan Benetti

**Affiliations:** University of Miami, 4600 Rickenbacker Causeway, Miami, FL 33149 USA

**Keywords:** Larviculture, Length-at-age, Mean daily growth increment, Von Bertalanffy Growth Model, Weight-at-age

## Abstract

Growth rates of larval and juvenile bigeye scad *Selar crumenophthalmus* reared in captivity were studied. The results are presented, discussed, and compared to wild *S. crumenophthalmus* and other pelagic fish. *S. crumenophthalmus* are a small pelagic carangid fish of circumtropical distribution. Larvae were reared in a modified mesocosm system and sampled daily for growth. Larvae grew to a mean size of 4.74 cm (Standard Length) and 1.30 g by 45 days post hatch (dph). Larval length-at-age was best described by the exponential equation *Y* = 1.966e^0.0704*t*^. For juvenile growth trials, 1940 fish were stocked into four 2.5 m^3^ cylindro-conical tanks at two different densities (262 fish m^-3^ and 120 fish m^-3^) and reared from 45 dph to subadult stage. Fish were sampled daily for growth. No statistically significant differences in growth or survival were detected between tanks. Mean length and weight at 141 dph was 13.24 cm (Total Length) and 25.20 g. Juvenile length-at-age was best described by the Von Bertalanffy Growth Model equation *L*_t_ *=* 27.75(1 – e^-0.03(t-1.57)^). Weight-at-age was best described by a linear equation *W*_t_ = 1.7313*x* + 12.4662. The exponent of the length-weight equation was 3.14. In addition to providing the first published description of larviculture and juvenile growout techniques for *S. crumenophthalmus*, this study contains the first published data on this species’ larval growth and directly confirms estimates of *S. crumenophthalmus* juvenile growth done by other researchers using indirect techniques such as otolith daily growth increment and frequency distribution analysis.

## Introduction

Bigeye scad *Selar crumenophthalmus* (Bloch) are a small pelagic, schooling fish of circum-tropical distribution (e.g. Dalzell and Penaflor 1989; Clarke and Privitera 1995; Mohammed and Rennie 2003; Hendiarti et al. 2005; Roos et al. 2007). Members of the *Carangidae* family, *S. crumenophthalmus* are an important forage fish for high trophic level predators, are highly valued as food in Asian and Pacific cultures, and are used as bait by recreational and commercial fishermen all over the world (Clarke and Privitera 1995; Honebrink 2000; Roos et al. 2007).

*S. crumenophthalmus* support a number of important fisheries throughout the world. In Hawaii more than 500 metric tons of *S. crumenophthalmus* (known locally as akule and hahalalu) are harvested each year for local consumption (Stevens 2004). In parts of the Indonesian and the Philippine archipelagos *S. crumenophthalmus* catches account for 3 to 15% of the annual small pelagic fish harvest (Dalzell and Penaflor 1989; Hendiarti et al. 2005). Around Reunion Island in the Indian Ocean, fishers take more than 35 metric tons of *S. crumenophthalmus* (known locally as atule) a year using hand lines and beach seines (Roos et al. 2007). In Florida and the Caribbean there is very little reliable data on the magnitude of *S. crumenophthalmus* (known locally as goggle eye) harvests but anecdotal evidence indicates that landings are significant, especially for use as live bait. In the Caribbean, live *S. crumenophthalmus* are the favored bait of the pelagic long line fishing fleet. Individual long line vessels take as many as 12,000 fish to sea per trip in large (~8500 l) live wells where they are used to target tunas (*Thunnus spp*.) and other high value fish (Capt. Don Landry, personal communication, 20 April 2010). In Florida, *S. crumenophthalmus* were formerly harvested as a food fish, largely for sale in the Asian market. The fishery, however, was based on unsustainable purse-seining practices and by the mid-1990s stocks had declined over large portions of the species’ range. As the stocks declined, the food-fish fishery was largely forgotten and the *S. crumenophthalmus* became increasingly valuable as live bait for recreational fishermen. Today *S. crumenophthalmus* in Florida retail for $ 100 for a dozen live fish or more (Capt. Butch Constable, personal communication, 15 October 2008).

Wild *S. crumenophthalmus* exhibit rapid growth rates, reaching a fork length (*L*_f_) of approximately 20 to 25 cm in one year (Dalzell and Penaflor 1989; Roos et al. 2007) and have been observed with a *L*_f_ of more 28 cm (Ralston and Williams 1988). They are partial spawners, and have been estimated to release 92,000 eggs per spawn (Clarke and Privitera 1995). In the wild, spawning occurs over an extended period during warm months (Kawamoto 1973; Tobias 1987; Clarke and Privitera 1995; Roos et al. 2007). *S. crumenophthalmus* are not generally believed to survive more than one spawning season, although a small number of animals are reported to live as long as three years (Kawamoto 1973; Ralston and Williams 1988). Feeding is largely nocturnal and planktonic animals such as euphasiids and fish larvae are favored prey items (Roux and Conand 2000).

In this paper the growth rates of larval and juvenile *S. crumenophthalmus* reared in captivity are compared with the growth rates of wild *S. crumenophthalmus* and other wild and hatchery-reared small pelagic fish, especially carangids. We also compare length-weight data for both wild and captive juvenile *S. crumenophthalmus* and describe larviculture and juvenile growout techniques for the species.

## Materials and methods

### Broodstock capture, maturation and spawning

All the fish used in this study were reared in captivity from eggs obtained from wild broodstock. All broodstock fish used for this study were captured in waters approximately five nautical miles east-southeast of Key Biscayne, Florida, in the vicinity of Fowey Rocks (National Data Buoy Center Station FWYF1), latitude 25.590 N 80.097 W and held at the University of Miami Experimental Hatchery (UMEH).

Broodstock fish were held in four 4.5 m^3^ cylindro-conical tanks connected to a single recirculating filtration system utilizing UV, mechanical (broken glass) and biological filtration. Each tank had its own egg collector. Broodstock fish were conditioned to spawn in their tanks by maintaining water temperature at 28 - 30°C, a level that corresponds to the upper end of the ambient temperature range experienced in south Florida coastal waters during the *S. crumenophthalmus* spawning season (NODC National Ocean Data Center 2010). Natural light was provided through a 95% reflective cloth. Water exchange was approximately 20% per day. Ammonia (NH_4_) was kept below 0.5 mg l^-1^. Dissolved Oxygen was maintained at or above saturation. Photoperiod was not manipulated.

The eggs used in this study came from two spawning events that were induced using hormone injections. The first group of fish (*n* = 20) was injected with Luteinizing Hormone-Releasing Hormone analogue (LHRH-a) at a dosage of 25 to 50 μg kg^-1^. The second group of fish (*n* = 20) was injected with Human Chorionic Gonadotropin (HCG) at a rate of 1000 IU kg^-1^. Both groups were segregated in separate maturation tanks. The fish were not sexed prior to injection. The fish injected with LHRH-a produced an estimated 54,400 eggs with a fertilization rate of 82.4% and an average diameter of 735 μm. The HCG injected fish produced an estimated 62,440 eggs with a fertilization rate of 96.7% and an average diameter of 744 μm. Eggs were pooled in a single incubator and the hatch rate was approximately 80%.

### Larval rearing

Approximately 71,000 yolk-sac larvae at 1 dph were stocked into a 1.8 m^3^ flat-bottomed larval-rearing tank supplied with 1 μm filtered seawater for this study. The early stage larval rearing protocols used in this study relied on a modified greenwater system adapted from other authors (Liao et al. 2001; Papandroulakis et al. 2002; Partridge et al. 2003; Palmer et al. 2007). A mixture of 50% *Isochrysis galbana* and 50% *Nannochloropsis oculata* was maintained in the larviculture tank at a density of ~400,000 cells ml^-1^. Due to a shortage of live algae, small amounts of commercially prepared non-viable *I. galbana* and *N. oculata* concentrates (NutrOcean, Inc., Rimouski, Quebec) were added on an as-needed basis to maintain the desired concentration of microalgae in the larval rearing tanks. The percentage of concentrate substitution never rose above 50%. Enriched s-type rotifers (*Brachionus rotundiformus*) were maintained in the tank at a density of 20–30 rotifers ml^-1^ via daily additions.

Beginning at 17 dph the culture was transitioned from a modified greenwater system to an intensive system adapted from Benetti et al. (2008). Water exchange was increased (ultimately reaching 1000% per day), enriched instar-2 *Artemia* were provided using a pulse feeding method, and algal and rotifer densities were allowed to drop to zero. Commercially prepared weaning feeds were provided along with the live feeds beginning at 22 dph. By 35 dph the fish were fully weaned onto commercially prepared feeds.

A 24-hour light photoperiod was used to rear the larvae through 6 dph using aquarium lighting. At 7 dph the photoperiod was changed to 18 hours light and 6 hours dark. Beginning at 16 dph the light period was reduced by one to one and a half hours hour per day until the photoperiod was entirely natural (~11 hours light and 13 hours dark) at 20 dph. Natural light was provided through an 80% shade cloth. Temperatures in the larviculture tank ranged from 25 to 28°C and dissolved oxygen (DO) levels were maintained above saturation (from 7 to 14 mg l^-1^) throughout the trial.

### Juvenile growout

At 45 dph all fish used for this study were transferred from their larval rearing tank to four 2.5 m^3^ cylindro-conical growout tanks. All four tanks were connected to a common recirculating filtration system utilizing UV, mechanical (broken glass) and biological filtration. Two tanks were stocked with 655 fish each (262 fish m^-3^) and two were stocked with 305 fish each (120 fish m^-3^). The mean length and weight of the fish stocked was 5.25 cm Total Length (*L*_t_) and 1.30 g in all tanks. Otohime EP1 and EP2 feeds (Aquatic Enterprise Co., Sarawak, Malaysia) were fed to the juvenile *S. crumenophthalmus* until 95 dph. Zeigler Marine Grower “Gold” 3.0 mm feed (44% crude protein, 18% crude fat) (Zeigler Bros, Inc., Gardners PA, USA) was provided until the end of this trial at four and a half months post hatch. Natural light was provided through a 95% reflective cloth. Oxygen was maintained at or above saturation for the duration of this trial. Temperature was maintained between 20 and 22°C, although on two occasions winter cold fronts caused water temperatures in the system to drop as low as 16°C. Water exchange was approximately 20% per day. Ammonia (NH_4_) was kept below 0.5 mg l^-1^. Fish were fed *ad libitum* twice a day. Aquaculture performance variables, including feed conversion rates (FCR), survival rates, and growth rates, were determined individually for each tank. The growout trial in the four 2.5 m^3^ tanks was ended at 141 dph because the filtration equipment was unable to handle the rapidly increasing bio-load and the fish were outgrowing the tanks. The fish were moved to a single, 12 m^3^ growout tank with flow-through, 10 μm filtered seawater.

### Data collection and analysis

10 larvae per day were randomly sampled for length from 3 to 24 dph (*n* = 220) when net avoidance behavior made it difficult to capture fish. Between 24 dph and 45 dph all samples were obtained by weighing and measuring otherwise healthy fish that died due to wall strikes (*n* = 14). This method of sampling was also used throughout the growout phase of the trial (n = 190). Fish that died of disease or unknown causes were not measured. This method of sampling was chosen because the events that triggered wall strikes were apparently random and because net avoidance behavior rendered it impossible to catch individual fish without creating excessive stress among the other fish in the tank. Additionally, fish were randomly sacrificed at 45 dph (*n* = 25) and 141 dph (*n* = 44) to supplement existing data and estimate mean size.

A group of wild juvenile *S. crumenophthalmus* (*n* = 33) was captured in order to establish a length-weight relationship that could be used as a baseline for comparison with captive-reared fish. These fish were captured using the same methodology that was used to capture broodstock, but were caught in shallow waters more immediately adjacent to Key Biscayne, Florida. Fish were stored on ice and transported back to laboratories at the University of Miami where they were weighed and measured. Fish were captured in July and September of 2009 and likely represent the result of spring and summer spawns from the same year.

A Von Bertalanffy Growth Model (VBGM) was developed in R (R Development Core Team, 2011) using the Fisheries Stock Assessment methods platform, a software toolbox for fisheries modeling (Ogle 2010). Best fit models for linear and non-linear relationships were developed and plotted using Newton least squares methods within R and Microsoft Excel 2010. Different mean growth rates and other aquaculture performance metrics between tanks were tested for statistical significance using a Students t-test with results considered significant at p ≤ 0.05. No outliers (runts) were excluded from the data set.

## Results

### Larval growth

**Figure 1:**
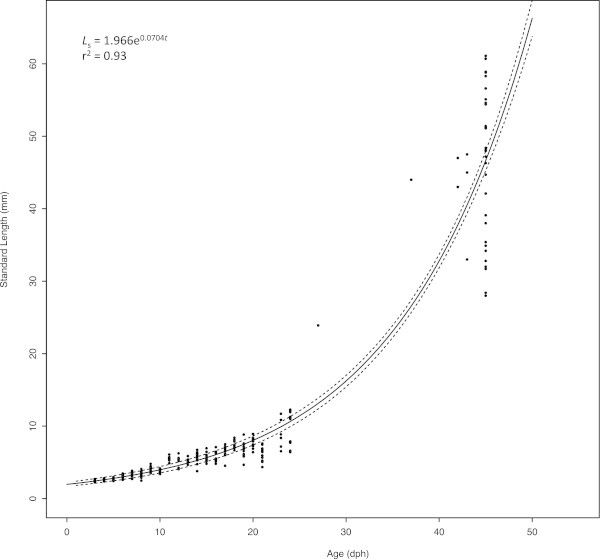
**The length-at-age, non-linear regression model (solid line) for** ***S. crumenophthalmus*** **larvae (n = 259) ages 1 – 45 dph.** Dashed lines represent the 95% upper and lower confidence intervals calculated using bootstrapped growth parameters. The model is based on Standard Length (*L*_s_).

Larval *S. crumenophthalmus* grew to a mean weight of 1.30 g and 4.74 cm Standard Length (*L*_s_) by 45 dph. Larvae had a mean length of 2.23 mm at hatching. Size heterogeneity within the cohort increased over time (Figure 1). The survival rate from yolk-sac larvae to 45 dph was approximately 2.74%. Metamorphosis was complete for all fish by 45 dph. Length-at-age (*L*_s_) to 45 dph was best described by the exponential equation

Mean daily growth for a given age was calculated by subtracting the mean hatch length (2.23 mm) from the mean length and then dividing by the relevant number of days. This resulted in a predicted mean Absolute Growth Rate (AGR) (mm day^-1^) of 1.03 mm day^-1^ (*L*_s_) to 45 dph (Table 1).

**Table 1:** **Growth rates for** ***S. crumenophthalmus*** **and selected carangid, clupeid, and engraulid larvae**

Species	Location	Growth rate	Reference
**Bigeye Scad**	Aquaculture	0.31 mm day^-1^ (*L*_s_) 1 to 24 dph	Current study
***Selar crumenophthalmus***		1.80 mm day^-1^ (*L*_s_) 24 to 45 dph	
		1.03 mm day^-1^ (*L*_s_) 1 to 45 dph	
**Horse Mackerel** ***Trachurus declivis***	Tasmanian Coast, Australia	0.24 - 0.29 mm day^-1^ (*L*_s_) to 15 dph	Jordan (1994)
**Rough Scad** ***Trachurus lathami***	Brazilian Bight, Brazil	0.41 mm day^-1^ (Body length) to 80 dph	Katsuragawa and Ekau (2003)
**Atlantic Bumper** ***Chloroscombrus chrysurus***	Southern Gulf of Mexico, Mexico	0.12 mm to 0.17 mm day^-1^ (*L*_s_) to 50 dph	Sanchez-Ramirez and Flores-Coto (1998)
**Jack Mackerel** ***Trachurus japonicus***	East China Sea, Japan	~ 0.6 mm day^-1^ (Body length) to 78 dph	Xie et al. (2005)
**Florida Pompano** ***Trachinotus carolinus***	Aquaculture	0.22 - 0.35 mm day^-1^ (*L*_f_), to 20 dph	Riley et al. (2009)
**Amberjack** ***Seriola dumerili***	Aquaculture	0.82 mm day^-1^ (*L*_t_), to 40 dph	Papandroulakis et al. (2005)
**Atlantic Menhaden** ***Brevoortia tyrannus***	North Carolina Coast, USA	0.22 – 0.35 mm day^-1^ (*L*_t_) to 20 dph	Warlen (1992)
**Japanese Anchovy** ***Engraulis japonicus***	Kuroshio Current, Japan	0.68 mm day^-1^ (*L*_t_) to 40 dph	Aoki and Miyashita (2000)
**Pacific Sardine** ***Sardinops sagax sagax***	Peru Current, Peru	0.7 - 0.8 mm day^-1^ (*L*_t_) to 15 dph	Butler and Rojas de Mendiola (1985)

**Figure 2:**
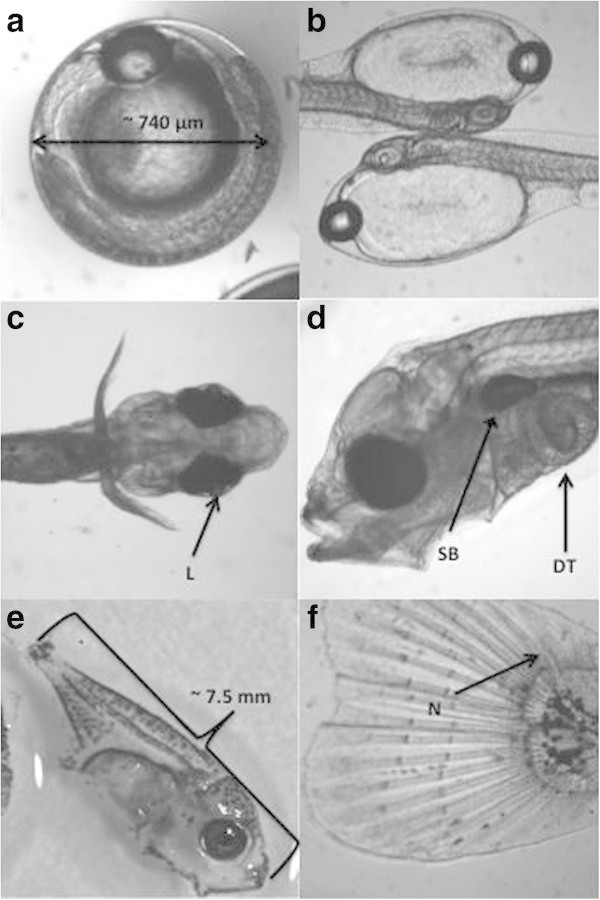
***S. crumenophthalmus*** **embryonic and larval development. (a-f)** Larval development of *S. crumenophthalmus*. **(a)** fertilized egg approximately 3 hours prior to hatching; **(b)** yolk-sac larvae at 1 dph; **(c)** larvae at 2 dph with prominent lens (L) readily visible; **(d)** larvae at 7 dph with prominent swim bladder (SB) and digestive tract (DT); **(e)** 18 dph post-flexion larvae; **(f)** tail of an 18 dph post-flexion larvae with upturned notochord (N).

Ontogenetic development proceeded rapidly (Figure 2). The embryonic yolk sac disappeared by the end of 1 dph and the associated oil globule was absorbed by 2 dph. Exogenous feeding and development of the very large lens that characterizes the eyes of adult *S. crumenophthalmus* was evident as early as 2 dph. Swim bladder development began between 4 and 7 dph. Digestive tract folding began at approximately 7 dph. Flexion began at 11 dph and was complete in all fish by 18 dph. Metamorphosis was complete for all fish by 45 dph, when the juveniles were transferred to growout tanks.

### Juvenile growth

Growth rates during the juvenile growout study (46 to 141 dph) were not significantly different in any of the tanks (p > 0.05). Fish grew to a mean size of 13.24 cm (*L*_t_) and 25.20 g by 141 dph. Specific growth rate (SGR) (% body weight day^-1^) was 3.08% based on mean weight at 141 dph. There were also no significant differences in aquaculture performance between the tanks (p > 0.05). FCRs in the tanks ranged between 1.30 and 1.62 with an overall FCR for the trial of 1.48. Survival rates in the tanks ranged from 88.1 to 92.1% with an overall mean survival rate for the trial of 89.1% (Table 2).

**Table 2:** **Selected growth and aquaculture performance parameters for captive juvenile** ***S. crumenophthalmus***

**Absolute growth (g)** *Δ*G = W_2_–W_1_	23.90
**Absolute growth rate (g day** ^**-1**^ **)** AGR = (W_2_–W_1_)/(t_2_–t_1_)	0.25
**Relative growth** RG = (W_2_–W_1_)/W_1_	18.38
**Relative growth rate** RGR = (W_2_–W_1_)/W_1_(t_2_–t_1_)	0.19
**Instantaneous growth rate (g day** ^**-1**^ **)** IGR = (lnW_2_–lnW_1_)/(t_2_–t_1_)	0.031
**Specific growth rate (g day** ^**-1**^ **)** SGR = 100 * (lnW_2_–lnW_1_)/(t_2_–t_1_)	3.08
**Mean weight (g) (± SD)** at 141 dph	25.20 (6.49)
**Mean length (cm) (** ***L***_**t**_ **) (± SD)** at 141 dph	13.24 (1.03)
**Feed conversion ratio** FCR = Feed offered/total biomass gain	1.48
**Survival (%)** from 46 to 141 dph	89.1

**Figure 3:**
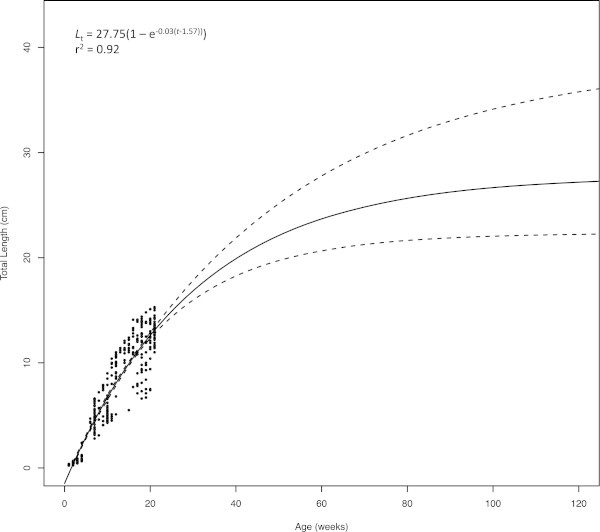
**Von Bertalanffy Growth Model (solid line) for** ***S. crumenophthalmus*** **(n = 493) with 95% upper and lower confidence intervals using bootstrapped growth parameters (dashed line) for age 1–21 week fish extrapolated to 120 weeks.** The model is based on Total Length (*L*_t_).

Because there were no statistical differences in aquaculture performance between tanks, growth curves and length-weight relationships were calculated using pooled data broken down into one-week (7 day) increments. We did not calculate separate curves for male and female fish because there was no basis for observing sexual dimorphism. Length-at-age for captive juvenile big eye scad was best described by the VBGM equation

(Figure 3). A linear relationship also provided a good fit for the length-at-age data and was described by the equation

**Figure 4:**
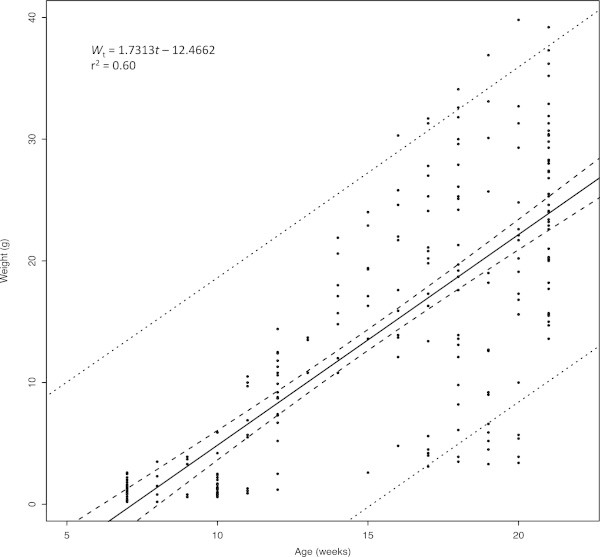
**Weight-at-age (** ***W***_**t**_ **) linear regression model (solid line) for** ***S. crumenophthalmus*** **(n = 259) for age 7 to 21 week fish with 95% upper and lower confidence bounds (dashed line) and 95% upper and lower prediction bounds (dotted line).**

**Figure 5:**
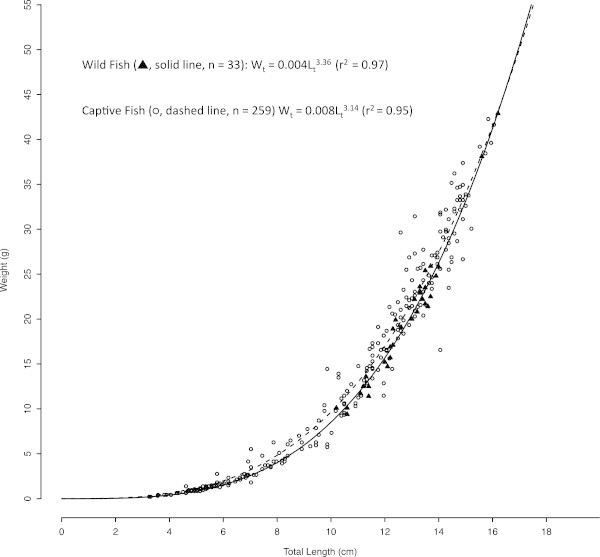
**Weight-at-length non-linear relationship of wild (**_**←**_ **, solid line, n = 33) and captive (○, dashed line, n = 259)** ***S. crumenophthalmus*** **.** The model is based on weight-at-age (*W*_t_) and total length (*L*_t_).

A linear relationship provided the best fit for the weight-at-age data and was described by the equation

(Figure 4). The length-weight relationship for captive reared *S. crumenophthalmus* was best described by the equation

(Figure 5). No statistically significant differences were noted between the length-weight relationships of wild and captive fish.

## Discussion

### Larval growth

The survival rate to 45 dph was relatively low (2.74%) in this study. Other species with more established larviculture technologies often have much higher survival rates over a comparable period of ontogenetic development. Cohorts of cobia (*Rachycentron canadum*) reared at the UMEH, for example, often exhibit survival rates of 15% or more (Benetti et al. 2010a). There is no published data on the growth rates of larval *S. crumenophthalmus* available in the scientific literature so there is no basis for comparing the growth of the larvae studied here with wild larvae. The larvae studied here, however, exhibited an AGR (mm day^-1^) greater than the reported growth rates of many other wild and hatchery-reared clupeid, engraulid and carangid species (Table 1). The growth rates of larval *S. crumenophthalmus* appeared to reach an inflection point around 24 dph. Up to 24 dph the mean AGR was 0.31 mm day^1^. Between 24 and 45 dph the rate increased to a mean of 1.80 mm day^-1^ (Figure 1). This increase in growth rate was accompanied by an increase in the swimming ability of the fish that was evidenced by the fact that sampling after 24 dph became extremely difficult due to net avoidance behavior. Similar improvements in the burst swimming speed of other species of pelagic fish larvae have been observed at comparable points in their ontogenetic development (Masuda 2006).

### Juvenile growth

Both a VBGM and a linear model provided a good fit for the data. The linear model provided a good fit for the data (r^2^ = 0.91) because the experiment ended at the sub-adult phase of development, before the asymptotic phase of the growth curve was reached. VBGM parameters were within the lower end of the range of values that have been estimated for wild *S. crumenophthalmus* by researchers using indirect sampling techniques such as otolith daily growth increment and frequency distribution analysis.

The VBGM for the captive reared *S. crumenophthalmus* studied here had an asymptotic length (*L*_*∞*_) of 27.75 cm (*L*_t_) and a coefficient of growth (*K*) of 0.03 week^-1^. Wild *S. crumenophthalmus* have been reported to have *L*_*∞*_ values ranging from 26.5 to 31.6 cm (*L*_t_) and *K* values ranging from 0.28 to 2.06 year^-1^ (Dalzell and Penaflor 1989; Garcia and Duarte 2006; Roos et al. 2007). Additionally, the VBGM developed here predicts that captive fish will reach a *L*_t_ of 14.9 cm at 6 months, 19.0 cm at 9 months, and 21.8 cm at one year. This is only slightly lower than the length-at-age values observed in the wild: Dalzell and Penaflor (1989) reported that *S. crumenophthalmus* in the Phillipines reach 23 cm (*L*_f_) in a year; Ralston and Williams (1988) report that Pacific Ocean *S. crumenophthalmus* attain 24.3 cm (*L*_t_) of growth by 330 dph; and Roos et al. (2007) report that Indian Ocean *S. crumenophthalmus* grow to 22.0 cm (*L*_f_) in one year.

Because temperature is known to be a major driver of fish growth and metabolism (Schmidt-Nielsen 1997) we believe that the temperatures maintained in the tanks (20-22°C) were suboptimal for juvenile *S. crumenophthalmus* growth and likely contributed to the lower growth rates. Even relatively small deviations from optimal temperatures can have large consequences for growth rates. Sun et al. (2006), for example, compared the growth rates of juvenile cobia (*Rachycentron canadum*) reared at 23 and 27°C and found that the fish reared at the higher temperature grew 44% faster. High stocking densities are also known to negatively affect the growth of cultured fish and may have been a contributing factor here (e.g. Faulk et al. 2007; Benetti et al. 2010b).

Even given their slower growth relative to their wild counterparts, the juvenile fish studied here exhibited a mean SGR (3.08%) that was higher than many other species of tropical and subtropical juvenile fish commonly reared in captivity. Cobia (*Rachycentron canadum*), mutton snapper (*Lutjanus analis*), amberjack (*Seriola dumerili*), and red drum (*Sciaenops ocellatus*), for example, exhibit SGRs ranging from 0.32 to 3.31% at comparable stages in their development (Skaramuca et al. 2001; Benetti et al. 2002; 2010b; Burr et al. 2006). Juvenile *S. crumenophthalmus* growth rates are not, however, extraordinary for tropical and subtropical pelagic fish. Juvenile mahi mahi (*Coryphaena hippurus*) cultured in captivity, for example, have been observed to have SGRs between 4 and 10% (Benetti et al. 1995).

Weight-at-age was difficult to describe mathematically due to the continued presence of very small fish (runts) in the tanks that appeared to weight the regression downward. Fish under 10 g were still being sampled as late as 140 dph. This resulted in considerable heterogeneity in the weight-at-age data. The best fit was a linear relationship (r^2^ = 0.60). This size-heterogeneity may have been the result of fish that were genetically predisposed to slow growth but were able to remain alive longer than they would have been able to as part of a wild population subject to size-specific mortality processes (Sogard 1997). Additionally, size-specific competition may have reduced access to feed for the smaller fish and further depressed their growth, especially in later stages of the growout trial.

The exponent of the length-weight equation (*b*) for captive *S. crumenophthalmus* was calculated to be 3.14. This is lower than the *b* values calculated for wild juvenile *S. crumenophthalmus*. The exponent of the length-weight equation generated from our sample of wild juvenile *S. crumenophthalmus* (n = 33) was 3.36 and Roos et al. (2007) found that the exponent of the length-weight equation ranged from 3.22 to 3.37 depending on the structure of the sample (male, female, or mixed). This was a surprising result. Captive reared fish tend to have a higher b value and condition factor than their wild counterparts, indicating that they are more robust and spherical in appearance and exhibit a more allometric growth pattern their wild counterparts (e.g. Blaxter 1988; Benetti et al. 1995; 2002; 2010b). The lower *b* value of the fish in this study departs from this trend. We believe that the continued presence of runts in the tank likely skewed this result downward.

Continued presence of runts is a characteristic of populations of fish reared in an aquaculture setting and this phenomenon was clearly seen here. Because many of the largest fish sampled in this trial, however, exhibited sizes that appeared more “normal” we believe that had the runts been removed from the data set as outliers we would have generated growth curves that more closely matched those presented by researchers working with wild fish.

## Abbreviations

AG: Absolute growth (*Δ*G = W_2_–W_1_)
AGR: Absolute growth rate (AGR = (W_2_–W_1_)/(t_2_–t_1_))
DO: Dissolved oxygen
Dph: Days post hatch
FCR: Feed conversion rate (FCR = Feed Offered/Total Biomass Gain)
HCG: Human chorionic gonadotropin
IGR: Instantaneous growth rate (IGR = (lnW_2_–lnW_1_)/(t_2_–t_1_))
Lf: Fork length
Ls: Standard length
Lt: Total length
L∞: Asymptotic length
LHRH-a: Luteinizing hormone-releasing hormone analogue
RG: Relative growth (RG = (W_2_–W_1_)/W_1_)
RGR: Relative growth rate (RGR = (W_2_–W_1_)/W_1_(t_2_–t_1_))
SD: Standard deviation
SGR: Specific growth rate (SGR = 100 * (lnW_2_–lnW_1_)/(t_2_–t_1_))
VBGM: Von Bertalanffy Growth Model
Wt: Weight-at-age.
